# A cutting-edge strategy for spinal cord injury treatment: resident cellular transdifferentiation

**DOI:** 10.3389/fncel.2023.1237641

**Published:** 2023-08-30

**Authors:** Yu-Ming Fang, Wei-Can Chen, Wan-Jing Zheng, Yu-Shen Yang, Yan Zhang, Xin-Li Chen, Meng-Qin Pei, Shu Lin, He-Fan He

**Affiliations:** ^1^Department of Anaesthesiology, The Second Affiliated Hospital of Fujian Medical University, Quanzhou, China; ^2^Centre of Neurological and Metabolic Research, The Second Affiliated Hospital of Fujian Medical University, Quanzhou, China; ^3^Neuroendocrinology Group, Garvan Institute of Medical Research, Sydney, NSW, Australia

**Keywords:** spinal cord injury, transdifferentiation, direct reprogramming, nerve repair, neurons

## Abstract

Spinal cord injury causes varying degrees of motor and sensory function loss. However, there are no effective treatments for spinal cord repair following an injury. Moreover, significant preclinical advances in bioengineering and regenerative medicine have not yet been translated into effective clinical therapies. The spinal cord’s poor regenerative capacity makes repairing damaged and lost neurons a critical treatment step. Reprogramming-based neuronal transdifferentiation has recently shown great potential in repair and plasticity, as it can convert mature somatic cells into functional neurons for spinal cord injury repair *in vitro* and *in vivo*, effectively halting the progression of spinal cord injury and promoting functional improvement. However, the mechanisms of the neuronal transdifferentiation and the induced neuronal subtypes are not yet well understood. This review analyzes the mechanisms of resident cellular transdifferentiation based on a review of the relevant recent literature, describes different molecular approaches to obtain different neuronal subtypes, discusses the current challenges and improvement methods, and provides new ideas for exploring therapeutic approaches for spinal cord injury.

## 1. Introduction

Spinal cord injuries (SCI) can be traumatic or non-traumatic. Traumatic SCI represent the most common type in clinical practice and is the focus of this review. Unlike the other regions of the adult brain, the spinal cord cannot produce new neurons during adulthood (Fan et al., 2022). Traumatic SCI leads to irreversible neuronal loss, impairing motor and sensory functions. Although research on the pathological mechanisms of SCI has progressed in recent years, clinical studies related to SCI repair have not yielded significant results. Therefore, the restoration of neurons after SCI is a key challenge.

Congenital neurogenetic processes are temporally and spatially limited under pathological conditions and are usually insufficient for functional neural repair. Therefore, neural regeneration must be promoted using exogenous factors to replace damaged and lost neurons. Biomaterial-based approaches mainly scaffolds, fibers, conduits, hydrogels, and sheets, hold therapeutic potential to promote axonal regeneration and motor function recovery (Ashammakhi et al., 2019). Specifically, Neuro-Spinal Scaffold shows positive results in clinical trials and holds promise for improving thoracic SCI (Theodore et al., 2016). Nevertheless, no biomaterial-based regenerative therapy of SCI has entered routine clinical practice to date, and extensive testing is still needed to demonstrate its safety and efficacy in patients. Likewise, therapies based on stem cell transplantation and induced pluripotent stem cell (iPSC) techniques are promising for regenerating new neurons after SCI (Csobonyeiova et al., 2019). However, recent studies have shown that transplant-based cell therapies face certain obstacles. The time required to prepare the cells for autologous transplantation is too long to provide an optimal time window in the subacute phase of SCI (Nishimura et al., 2013). Next, iPSCs must be extensively expanded and differentiated in culture before transplantation, which is time-consuming (Okano et al., 2013).

Compared to the above approaches, an emerging regenerative strategy involves the direct transformation of mature terminally differentiated cells into functional neurons in the mammalian central nervous system (Li and Chen, 2016). This process of transferring somatic cells from one lineage to another without undergoing an intermediate pluripotent state is described as “direct reprogramming” or “transdifferentiation” (Wang H. et al., 2021). The transdifferentiation approach is faster, more efficient, and safer because it does not involve the intermediate stages of iPSC generation and excludes the possibility of iPSC-related tumorigenesis (Lee et al., 2013). Furthermore, compared with indirect reprogramming, transdifferentiation can occur *in situ* and is more suitable for *in vivo* tissue repair. This strategy inherits the unique age-related characteristics of transdifferentiated neurons and provides an advantage in mimicking the cellular processes that occur with aging (Tang et al., 2017; Cates et al., 2021). Major neuronal transdifferentiation methods have been established to date that allow the direct conversion of somatic cells into inducible neurons (iNs). Proliferating neural precursor cells, such as induced neural progenitor cells or iNPCs, can also be derived using these methods (Mollinari et al., 2018). We hypothesized that regenerative medicine based on cell fate transformation has great potential for treating SCIs with the emergence of new evidence.

However, our review is not the first to report the application of neuronal transdifferentiation in SCI. Compared to previous reports on somatic cell reprogramming techniques for the treatment of SCI (Yang et al., 2022), we summarize the current state-of-the-art in transdifferentiation of human cells and resident cell transdifferentiation in the injury microenvironment *in vivo*. As the mechanism behind transdifferentiation is currently unclear, we have facilitated the optimization of the results of transdifferentiation schemes by dissecting and comparing the transdifferentiation mechanisms of different approaches. More importantly, we searched for appropriate transdifferentiation protocols in the direction of neuronal subtypes, which can effectively complement the specific functional neuronal subtypes of injuries. The aim is to accelerate the clinical translation of these directly transformed neuronal cells to treat SCI.

## 2. *In vitro* neuronal transdifferentiation is fundamental

Transdifferentiation is achieved mainly by the ectopic overexpression of specific TFs. A group of transdifferentiating factors, including OCT4, SOX2, KLF4, and c-MYC (also known as direct reprogramming factors), were first reported to directly reprogram fibroblasts into functional neural precursor cells (Kim et al., 2011). Subsequently, Vierbuchen et al. (2010) discovered that a combination of three factors, Ascl1, Brn2, and Myt1l, was sufficient to rapidly and efficiently convert mouse embryonic and postnatal fibroblasts into functional neurons *in vitro* and that the iNs generated action potentials and formed functional synapses. Transdifferentiating factors, such as Sox2 (Wang et al., 2016), Ascl1 (Liu et al., 2015), NeuroD1 (Guo et al., 2014), and NeuroG2 (Liu et al., 2021), have been explored in studying *in vitro* neuronal cell transdifferentiation. Forced expression of these TFs has effectively converted different cell types into functional neurons *in vitro*. In addition, microRNAs (miRNAs) and small molecules, such as miR-9/9*-124, can induce neuronal transdifferentiation and are effective transdifferentiating effectors, inducing the conversion of fibroblasts to cultured neurons (Yoo et al., 2011), together with valproic acid (VPA), CHIR99021, RepSox, Forskolin, SP600125, GO6983, and Y-27632 (VCRFSGY) or Forskolin, ISX9, CHIR99021, I-BET151, and SB43154 (FICBS), a cocktail of small molecules, reprogrammed fibroblasts into functional neurons. Both chemical cocktails induced neurons to achieve the basic goal of extending neurites and electroactive cells (Babos and Ichida, 2015; Hu W. et al., 2015). Generally, *in vitro* transdifferentiation approaches could be used to explore possible therapeutic targets and target factors for neurological disorders.

In most reports on transdifferentiation, transdifferentiated cells are obtained *in vitro* and transplanted into animals to evaluate their physiological functions. These cells are usually a single-cell type, and the transformation of these cells occurs in a controlled environment. In contrast, *in vivo*, transdifferentiation usually occurs in the injured microenvironment, where the surrounding cellular and molecular environment varies depending on the type of injury or neurodegenerative disease. Additionally, combinations and interactions between different cell types exist *in vivo*. Each cell type responds specifically to a given set of injury conditions, posing a challenge for *in vivo* transdifferentiation (Gascón et al., 2017). However, transdifferentiation strategies can still induce neurons *in vivo* (Torper et al., 2013; Pereira et al., 2017).

## 3. *In vivo* neuronal transdifferentiation becomes a new tool

There is growing evidence that the central nervous system is more plastic than previously thought and may contain a reservoir of cells with potential neurogenic programs *in vivo*. Therefore, we explored the generation of new neurons *in vivo* through cellular transdifferentiation, replenishing damaged or lost neurons and reconstructing the function of the damaged nervous system, including that of SCI. Here, we discuss TFs, miRNAs, and small-molecule-mediated transdifferentiation *in vivo*. We outline some studies on factor- and small-molecule-mediated neuronal fate transformation to identify the relevant neuronal transdifferentiation (Table 1).

**TABLE 1:** Summary of direct neural transdifferentiating factors/molecules and outcomes *in vivo*.

Transdifferentiating factors/Molecules	Delivery method	Starter cell	Targeted cells	Region *in vivo*	Derived neurons’ function	References
Sox-2	Lentivirus	Astrocytes	Neuroblasts	Spinal cord	N/D	Su et al., 2014
Sox-2 + VPA	Lentivirus	Astrocytes	GABAergic interneurons	Spinal cord	Axon regeneration; Integrating into the neural circuitry	Su et al., 2014
Sox-2 + p53-p21KO + BDNF + NOG	Lentivirus	Astrocytes	Glutamatergic neurons (>80%)	Spinal cord	Integrating into neural circuitry (Synaptic connections)	Wang et al., 2016
Sox-2	Lentivirus	NG2 glial cell	NPCs	Spinal cord	Integrating into neural circuitry; Reducing scar tissue	Tai et al., 2021
Ascl1 + Brn2 + Myt1l(ABM)	Lentivirus	Astrocytes	Dopaminergic neurons	Striatum	N/D	Torper et al., 2013
Ascl1 + Lmx1a + Nurr1	AAV	NG2 glial cell	Fast-spiking parvalbumin-containing Interneurons	Striatum	Integrating into the neural circuitry	Pereira et al., 2017
NeuroD1	Retrovirus	Reactive glial cells	Glutamatergic neurons	Cortex	Integrating into the neural circuitry	Guo et al., 2014
NeuroD1	Retrovirus	NG2 glial cell	Glutamatergic and GABAergic neurons	Cortex	Integrating into the neural circuitry	Guo et al., 2014
NeuroD1 + Nurr1	AAV	Astrocytes	Pyramidal neuron	Cortex	Integrating into the neural circuitry	Mattugini et al., 2019
NeuroD1	AAV	Astrocytes	Pyramidal neuron (90%)/GABAergic neurons (10%)	Motor cortex	Integrating into neural circuitry; Reducing inflammation	Chen et al., 2020
NeuroD1	AAV	Astrocytes	Glutamatergic (major)/GABAergic↑ by NeuroD1 + Dlx2	Spinal cord	Integrating into the local spinal cord functional circuitry	Puls et al., 2020
NeuroG2	AAV	Astrocytes	Glutamatergic and GABAergic neurons	The spinal cord, Dorsal midbrain	Integrating into the neural circuitry	Liu et al., 2021
NeuroG2 + Isl1	AAV + dCas9-VP64 + sgRNAs	Astrocytes	Motor neurons	The gray matter of the spinal cord	Projecting into the sciatic nerve to innervate skeletal muscles	Zhou et al., 2021
miR-302/367	Lentivirus	Astrocytes	Neuroblasts	Striatum	Reducing scar tissue	Ghasemi-Kasman et al., 2015
miR-302/367 + VPA	Lentivirus	Astrocytes	Myelinating cells/OPCs	Demyelinated corpus callosum	Remyelination	Ghasemi-Kasman et al., 2018
FICBY	osmotic mini-pumping system	Astrocytes	GABAergic neurons (87.1%)	Striatum	Integrating into the neural circuitry	Ma et al., 2021
FICBY	osmotic mini-pumping system	Astrocytes	Glutamatergic neurons (72.8%)	Cortex	Integrating into the neural circuitry	Ma et al., 2021

AAV, Adeno-associated virus; FICBY, Forskolin + ISX9 + CHIR99021 + I-BET151 + Y-27632; N/D, no data (experiment not done); NPCs, Neural progenitor cells; OPC, Oligodendrocyte progenitor cells; PTB, Polypyrimidine tract-binding.

### 3.1. Transcription factor-mediated transdifferentiation

Overexpression of key TFs has been used for many years to successfully transform various cell types *in vivo*. Glial cells, including astrocytes and NG2 glial cells, account for most *in vivo* transdifferentiation. SOX-2 first transforms resident astrocytes in the adult spinal cord into doublecortin (DCX)-positive neuroblasts, which require further treatment with the histone deacetylase inhibitor VPA to differentiate into mature neurons (Su et al., 2014). In a subsequent study, SOX-2-induced transdifferentiation significantly increased induced adult neuroblasts (iANBs) production by silencing the p53 pathway (Wang et al., 2016). Although the number of induced cells is high, fertility affects the chromatin status of specific genes involved in the transformation (Cates et al., 2021). Contrarily, Ascl1-induced cell cycle arrest improved neuronal transdifferentiation. Ascl1 is specifically expressed in astrocytes by infusing a glial fibrillary acidic protein (GFAP)-adeno-associated virus (AAV) vector into the dorsal midbrain, and astrocytes in the striatum and somatosensory cortex are transformed into functional neurons *in vivo* (Liu et al., 2015). An optimized combination of factors (Ascl1, Brn2, and Myt1l) was synergistically expressed in a combinatorial approach for the better transdifferentiation of mature neurons *in situ* (Torper et al., 2013).

In other studies, Brulet et al. (2017) found that NeuroD1 induces the conversion of astrocytes to neurons (ATN) under physiological conditions, thus facilitating functional recovery from SCI long after the acute injury stage. Subsequent studies further showed that other TFs, namely, NeuroG2, Isl1, Lhx3, Lmx1a, and Nurr1 (Mazzoni et al., 2013; Torper et al., 2015; Liu et al., 2021; Zhou et al., 2021), could also reprogram glial cells directly into functional neurons *in vivo*. Notably, NeuroG2 can transform astrocytes in the damaged spinal cord into functional neurons (Liu et al., 2021). Therefore, TFs are the main regulators of induced cellular transformation, and introducing exogenous genes into cells using viral vectors represents the main method (Parr-Brownlie et al., 2015). Among viral vectors, the expression of AAV-mediated factors has a slow onset of action and kinetics. This helps avoid the early inflammatory response induced by injury and cellular transformation that occurs after reducing the inflammatory environment (Torper et al., 2015). In addition, AAV was safe and efficacious for spinal cord vector delivery (Bravo-Hernandez et al., 2020). These advantages make AAV the best choice among many viral vectors for clinical use.

Interestingly, studies identified more TFs with the ability to induce transdifferentiation. CRISPR-activated strategies have been used to specifically identify loci with associated epigenetic markers to discover and activate the expression of endogenous transdifferentiation factors (Konermann et al., 2015; Black et al., 2016). Furthermore, CRISPR/Cas9 gene editing can be used to upregulate or silence the expression of relevant TFs to achieve accurate transdifferentiation (Chavez et al., 2015). For example, Cas9 can activate endogenous Brn2 and Ngn1, leading to efficient transdifferentiation of fibroblasts into neurons (Liu et al., 2018).

Mogrify is a computational framework that uses network biology to predict the combination of TFs required for direct transformation between human cell types; it assesses the ability of each TF to determine the fate of the starting and target cell types, thus enabling the identification of TFs located at the top of the gene regulatory network (D’Alessio et al., 2015; Rackham et al., 2016). Therefore, new technologies, such as CRISPR-Cas9 screens and computational biology, can be used to predict the transdifferentiating factors required to transform other cells.

### 3.2. miRNA-mediated direct cellular transformation

miRNA-mediated transdifferentiation involves two pathways. One is a pathway in which miRNA-rich neurons repress non-neuronal transcription, while the other activates repressed neural TFs in non-neuronal cells, both creating a favorable environment for the expression of neuronal gene programs (Adlakha and Seth, 2017).

Several neuron-specific miRNAs, such as miR-9/9* and miR-124, play key roles in transdifferentiating fibroblasts into functional neurons (Yoo et al., 2011). Further studies revealed that the inhibition of miR-124-targeted polypyrimidine tract-binding protein (PTB or PTBP1) and its analog, nPTB (PTBP2), in different cell types *in vitro* induces the direct transformation of cells into functional neurons (Xue et al., 2013). Moreover, miR-124 directly targets and inhibits PTBP1 during development (Makeyev et al., 2007), showing that the interaction between miRNA and PTB may play an important role in neuronal transdifferentiation. Interestingly, PTBP1 inhibition induces ATN transformation (Zhou et al., 2020). Thus, miR-9/9*-124 may ameliorate SCI *in vivo* by acting as an effector of neuronal transdifferentiation. In an *in vivo* study, miR-302/367 induced a high conversion of astrocytes to neuroblasts upon co-administration with VPA (Ghasemi-Kasman et al., 2015). Although astrocytes are neuronally transformed by default in the demyelinated brain, they may change their fate to oligodendrocyte precursor cells (OPC) and myelin cells (Jablonska et al., 2010). Subsequent studies have shown that the forced expression of miR-302/367 clusters and astrocyte administration of VPA increased the likelihood that the demyelinated corpus callosum repairs myelin injury via astrocyte-generated oligodendrocytes (Ghasemi-Kasman et al., 2018). Thus, miR-302/367-mediated transdifferentiation may promote myelin regeneration in injured spinal cords.

### 3.3. Direct transdifferentiation induced by small molecules

Although TF-based genetic approaches are widely studied, it is impossible to avoid virus-based TF delivery because even the most promising AVVs are at risk of genetic mutations, and worse, we cannot avoid the possible toxicity of AVV titers (Bravo-Hernandez et al., 2020). Significantly, none of the transdifferentiation factors involved in the paper were found to be tumorigenic in their experiments. However, we cannot ignore that individual TFs are also highly expressed in tumor tissues (Porter and McCaughan, 2020). In miRNA-mediated transdifferentiation, it remains elusive because of the large number of miRNA complementary targets in the genome and the complexity of miRNA gene regulation. Moreover, PTBP1, as one of the main mechanisms mediating miRNA transdifferentiation, has faced many controversies in recent years, which is an obstacle to further application of miRNA-mediated transdifferentiation (Wang et al., 2021; Hoang et al., 2022). However, compared to TFs and miRNAs, small molecules are non-immunogenic and not integrated into the genome; moreover, their manipulation of intracellular targets is reversible (Federation et al., 2014). They have a high level of cell permeability, are easy to synthesize and standardize, and are suitable for mass cell production (Xu et al., 2015). Next, using viral vector-mediated genetic transdifferentiation has significant safety risks, such as the integration of vector genes into the human genome leading to tumorigenesis (Lu et al., 2013). Hence, a small molecule-mediated introduction instead of a viral vector-mediated approach can avoid reintroducing exogenous genes used in transdifferentiation (Son et al., 2011). Last but not least, the efficiency of transdifferentiation and the number of transdifferentiated neurons is usually very low. By analyzing the content of related studies (Liu et al., 2016; Rivetti di Val Cervo et al., 2017), we speculate that applying some small molecules may improve the safety and efficiency of transdifferentiation.

Hu W. et al. (2015) demonstrated that human dermal fibroblast-induced neurons from patients with Alzheimer’s disease showed neuronal characteristics similar to those of normal, directly reprogrammed cells, thus identifying new ideas for applying chemically induced transdifferentiation approaches to neurological diseases. Although few reports indicated that small molecules could induce neuronal transdifferentiation *in vivo*, the advantages and potential of small molecules for cell fate manipulation cannot be denied. In a strong test of *in vivo* chemical reprogramming, Yin et al. (2019) unexpectedly found that a mixture of small molecules (DAPT, CHIR99021, SB431542, and LDN193189) was able to significantly accelerate neuronal maturation and adult neurogenesis using intracranial or intraperitoneal injections. Nonetheless, this test does not exclude that the induced neurons originated from other resident cells, and further study is needed to show stable induction of small molecule-mediated direct reprogramming *in vivo*. Specifically, Ma et al. (2021) demonstrated that endogenous astrocytes could be induced into neurons in the mouse brain using a cocktail of small molecules (Forskolin, ISX9, CHIR99021, I-BET151, and Y-27632; FICBY). FICBY synergistically reprogrammed astrocytes in the cortex or striatum directly into chemically induced neurons (ciNs), which showed the ability to connect to endogenous neurons. This study strongly demonstrates the role of small molecule-mediated transdifferentiation in the nervous system, and motor neurons (MNs) are an extremely important type of neuron residing in the spinal cord. Research developed a small molecules cocktail (Kenpaullone, Forskolin, Y-27632, Purmorphamine and Retinoic acid; KFYPR) that induced the formation of MN-like cells from resident spinal cord astrocytes *in vivo*. Since astrocyte activation after SCI results in a glial scar that inhibits motor neuron, the direct conversion of resident reactive astrocytes into MN-like cells would promote functional recovery from SCI (Bradbury and Burnside, 2019; Zhao et al., 2020). These data show that *in vivo* chemically mediated transdifferentiation is a potential method for neuronal function compensation following neural injury. However, no technology is perfect, and off-target effects during induction and potential toxicity to other neuronal cells have not been extensively studied (Langie et al., 2015; Ma et al., 2021). Therefore, in the future, an ideal approach should be explored to deliver small molecules safely and effectively without causing invasive damage to the organism, and to reduce off-target effects to facilitate further applications of chemically mediated transdifferentiation in neural regeneration.

## 4. Study of molecular mechanisms of neuronal transdifferentiation

**FIGURE 1:**
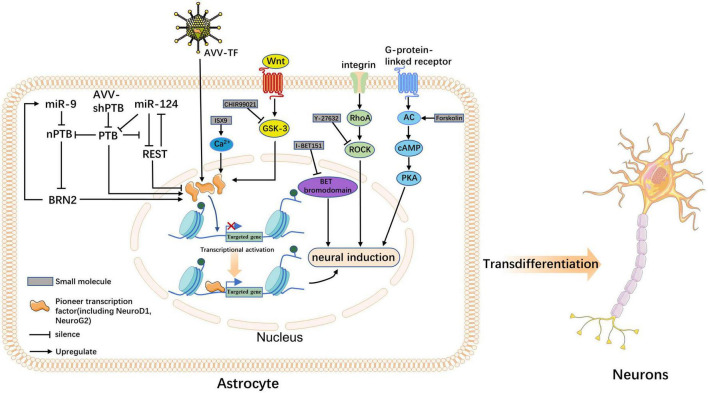
The molecular mechanism underlying neuronal transdifferentiation. This figure summarizes how transcription factors (TFs), microRNAs (miRNAs), and five small molecules acting *in vivo* induce astrocyte fate switching; the outcome is the direct transdifferentiation of astrocytes into neurons. Precursor TFs increase the accessibility of otherwise silenced target genes, making them key hubs for the three classes of actors. TFs and miRNAs act as endogenous factors to induce the overexpression of precursor factors through *ex vivo* pathways, activating the transcription of neuronal programs. Small molecules can also play a role in inducing transdifferentiation through transcription factor-mediated pathways; however, the mode of action of small molecules is more likely to promote neuronal fate through signaling pathways such as Wnt, ROCK, and cyclic AMP-protein kinase A.

Although several cellular transdifferentiation strategies have been established, their underlying molecular mechanisms remain unclear. However, recent advances in single-cell RNA sequencing (RNA-seq) techniques (Rao et al., 2021), *in vivo* time-delayed cell imaging, and real-time quantitative reverse transcription polymerase chain reaction (Aravantinou-Fatorou et al., 2015; Abernathy et al., 2017) can help reveal underlying molecular mechanisms. Often, the reprogramming of extracellular cues can lead to the induction of intracellular forces, such as TFs, chromatin modifications, and signaling pathways, which promote cell fate conversion. Next, we elaborate on the possible mechanisms by which TFs, miRNAs, and small molecules regulate transdifferentiation (Figure 1).

### 4.1. Transcription factors and miRNAs are endogenous epigenetic regulators

#### 4.1.1. Epigenetic modification: chromatin accessibility

Although the induction of transdifferentiation in the injured environment requires the involvement of endogenous factors (TF or miRNAs), ectopic expression of these factors is also necessary to unleash the full neurogenic potential of endogenous glial cells (Zong et al., 2016; Tai et al., 2021). Notably, pioneer TFs are considered major regulators and determinants of spectrum fate. Their structural domains can interact directly with chromatin to open the closed conformation of DNA and initiate transcriptional activation, while inducing other TFs to bind to DNA and participate synergistically in transcriptional regulation (Iwafuchi-Doi and Zaret, 2014, 2016). For example, reprogramming fibroblasts into iNs using Ascl1, Brn2, and Myt1l, Ascl1 acts as a pioneer factor, binding to physiological targets, even in closed chromatin regions, to activate transcription and actively recruit other TFs to some of its targets (Vierbuchen et al., 2010; Wapinski et al., 2013). Interestingly, ASCL1 induces rapid transcriptional changes and promotes an increase in the histone activation marker H3K27ac (the acetylation of histone 3 at the lysine 27 position) to many binding loci (Aydin et al., 2019). H3K27ac is a histone acetylation modification positively correlated with gene expression. The pioneering factor activities of NeuroG2 and NeuroD1 have also been confirmed in other studies (Pataskar et al., 2016; Smith et al., 2016). These factors are included in most neuronal transdifferentiation protocols, leading to increased accessibility of silent DNA and increased epigenetic H3K27ac histone markers. They may be common mechanisms for pioneer factor-mediated transcriptional regulation of different cell types (Aydin et al., 2019), allowing downstream effectors to bind and activate the transcription of neuron-specific genes to maintain their transition to neurons further, thus facilitating their role in epigenetic modifications involved in transcriptional regulation and epigenetic modifications involved in cellular transdifferentiation (Zaret and Carroll, 2011; Raposo et al., 2015).

In most cases, miRNAs do not act alone, but in concert with different TFs or controller elements to create the distinct network required for transdifferentiation. For example, miR-124 alone is required in concert with TFs to induce neuronal transdifferentiation (Ambasudhan et al., 2011). We found that miRNAs also appeared to reprogram cells by inducing epigenetic changes. Ectopic expression of miR-9/9*-124 during the induction of neuronal transdifferentiation coordinates the reduction of repressor element-1 silencing transcription factor (REST) protein stability; furthermore, it disrupts its expression, inducing chromatin accessibility and DNA methylation remodeling, thereby promoting gene expression (Abernathy et al., 2017; Lee et al., 2018). In summary, many studies have shown that miRNAs are potent regulators of cell fate and epigenetic regulators necessary for chromatin remodeling (Gruber and Zavolan, 2013).

#### 4.1.2. miRNA-PTB-REST regulatory loops

**FIGURE 2:**
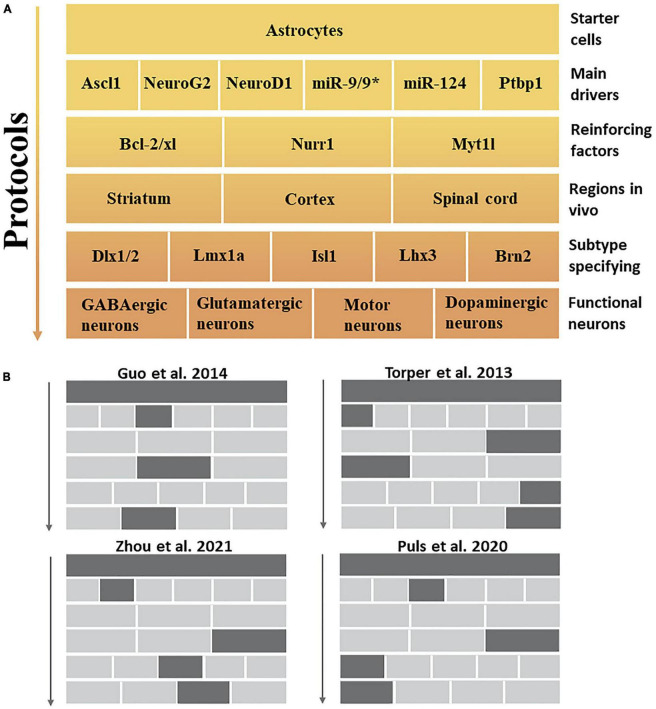
*In vivo* protocol for the induction of specific neuronal subtypes. **(A)** This figure summarizes the aspects to be considered in transdifferentiation protocols that describe the top-down pathway for generating specific neuronal subtypes. The main drivers and reinforcing factors act as public players in most approaches, primarily inhibiting non-neuronal fates and cellular transformation to pan-neuronal fates. Meanwhile, different brain regions and subtype specification factors further formulate specific functional neuronal subtypes. **(B)** Examples of experiments that induce different neuronal subtypes *in vivo* mentioned in this review.

PTB (PTBP1) is an RNA-binding protein mainly involved in RNA metabolism and plays a key role in neurogenesis (Xue et al., 2009). Inhibition of PTBP1 has been shown to directly transform fibroblasts into functional neurons in culture (Xue et al., 2013). Qian et al. (2020) based on this discovery, successfully demonstrated that knockout of PTBP1 alone could transform mouse astrocytes into mature functional neurons *in vivo*, and the transdifferentiation efficiency reached 80%. Thus, overcoming this obstacle could effectively improve the outcomes of transdifferentiation. A key event in the downregulation of glial cell-to-neuron transformation induced by PTBP1 is the inhibition of gene regulatory loops. Figure 2 shows the stable downregulation of PTBP1 prioritizes activating neuronal TFs and brain-specific miRNAs while downregulating anti-neuronal proteins, such as REST; moreover, it inhibits the expression of many neuro-specific TFs and miR-124 and acts as a transcriptional repressor, which constitutes a significant obstacle in the process of neuronal transdifferentiation. It should be emphasized that REST and PTB proteins are also regulated by miR-124 and miR-9/9* (Xue et al., 2013; Cusanovich et al., 2018). Generally, these findings show that the downregulation of PTBP1 induces a higher intensity of neuronal expression by amplifying the miRNA-PTB-REST regulatory loop.

In another part of the loop, when PTBP1 levels are increased, the paralog nPTB is downregulated, and nPTB controls mRNA splicing in newborn neurons (Makeyev et al., 2007). Further downregulation of nPTB increases the transcriptional activator BRN2 levels, which targets key TFs, thus promoting neuronal maturation. In this regulatory loop, BRN2 transcription activates miR-9, which in turn reduces nPTB levels after transcription. These data showed that nPTB knockout combined with BRN2 expression was sufficient to induce functional neurons (Xue et al., 2016). According to a recent study, AAV-shRNA- and ASO-mediated PTB knockouts can supplement motor neuron-like cells around the injured area and improve the recovery of motor function by reducing the density of glial scarring in a mouse model of SCI (Yang et al., 2023). Therefore, reducing PTB expression by modulating the miRNA-PTB-REST loop and thus PTB expression may be a promising therapeutic strategy for treating SCI.

Although TFs and ptbp1 silencing-mediated transdifferentiation are a research hotspot, there are questions about their real effects and reliability. Through our above exploration of their mechanisms, combined with recent studies (Wang et al., 2021; Chen et al., 2022; Xie et al., 2022), the skepticism that induced neurons may not be derived from astrocytes *in situ* has ensued. For example, while NeuroD1 and shPTB can have a positive effect on disease function improvement, by lineage tracing YFP-expressing astrocytes, it was surprising to find that AAV-mediated NeuroD1 or shPTB did not provide evidence of astrocyte-to-neuron conversion. Moreover, it was found that the overall neuronal density remained unchanged. Thus, a possible causal mechanism for misdirecting the origin of the neuron is that the GFAP promoter cell specificity can be altered by either a trans or cis mechanism of downstream factors or expressed genes induced by NeuroD1 or shPTB. Specifically for PTBP1, the authors did not observe a significant reduction in endogenous PTBP1 expression when using CRISPR-CasRx knockdown in mouse cortex, nor did they observe glial cell transdifferentiating into neurons during lineage tracing; And when knockdown was performed using shRNA, although endogenous PTBP1 knockdown, no transdifferentiation of genealogically labeled astrocytes into neurons was observed (Wang et al., 2021). The discussion of PTBP1 continues and warns that future experiments should explore transdifferentiation strategies more cautiously.

### 4.2. Small molecules and neuronal transdifferentiation

Small molecules induce neuronal transdifferentiation by interfering with signaling pathways and epigenetic regulation (Table 2). Consistent with previous studies, cocktails of small molecules containing VPA, CHIR99021, Repsox, Forskolin, SP600125, GO6983, and Y-27632 coordinated multiple signaling pathways, such as Wnt, TGF-β, cyclic AMP (cAMP), Notch, GSK-3, and BMP-mediated signaling pathways. These seven small molecules downregulate fibroblast-specific genes and increase the expression of endogenous neuronal TFs, thereby promoting neuronal cell transformation (Hu W. et al., 2015). In addition, forskolin and dorsomorphin can alter the epigenetic status by increasing chromatin accessibility (Smith et al., 2016). These data show that small-molecule compounds induce a more adaptive state of cell fate transition through signaling pathways regulation and epigenetic modifications. Hence, to reveal the mechanisms of endogenous cell fate transformation induced by small molecules *in vivo*, the researchers analyzed the coordination of ATN transformation induced by a combination of five small molecules (FICBY) (Ma et al., 2021). Forskolin promoted the efficiency of neuronal transformation by alleviating oxidative stress, combined with other related reports (Gascón et al., 2016). Hu W. et al. (2015) found that the induction ability of forskolin may be related to its mechanism of activating adenylate cyclase (AC). AC increased cAMP levels. In the same study, researchers found that glycogen synthase-3 (GSK3) signal transduction may be an obstacle to neuronal fate and that CHIR99021 was able to overcome this obstacle. CHIR99021 is an inhibitor of GSK3, which activates the Wnt signaling pathway by inhibiting GSK3 (Clevers and Nusse, 2012). Interestingly, inhibition of GSK3 signaling also improved neuronal transformation of human fibroblasts stably transduced by pioneer factors Ascl1 and Ngn2 and promoted neural induction (Ladewig et al., 2012; Li et al., 2012). Notably, a variety of small molecular cocktails, including forskolin and CHIR99021, show that glycogen synthase kinase-3 inhibition and cAMP stimulation play a key role in neuronal induction (Babos and Ichida, 2015).

**TABLE 2:** Most common small molecules in direct neuronal transdifferentiation and their action on the target pathways.

Small molecules	Main function	Transdifferentiated cell	References
Valproic acid (VPA)	Histone deacetylase inhibitor	Neural progenitor cells	Cheng et al., 2014
		Functional neurons	Hu W. et al., 2015; Zhang et al., 2015
RepSox	TGF-Beta inhibitor	Neural progenitor cells	Cheng et al., 2014
		Functional neurons	Hu W. et al., 2015
SP600125	JNK inhibitor	Functional neurons	Hu W. et al., 2015
GO6983	PKC inhibitor	Functional neurons	Hu W. et al., 2015
Dorsomorphin	BMP and TGF-Beta inhibitor	Functional neurons	Liu et al., 2013; Hu W. et al., 2015
Forskolin	Adenylyl cyclase activator	Neural progenitor cells	Cheng et al., 2014
		Functional neurons	Liu et al., 2013; Hu W. et al., 2015; Li et al., 2015
ISX9	Ca^2+^ signaling activator	Functional neurons	Li et al., 2015
CHIR99021	GSK-3 inhibitor	Neural progenitor cells	Cheng et al., 2014
		Functional neurons	Hu W. et al., 2015; Zhang et al., 2015
I-BET151	BET bromodomain inhibitor	Functional neurons	Li et al., 2015
Y-27632	Rock inhibitor	Functional neurons	Hu W. et al., 2015
TTNPB	Retinoic acid activator	Functional neurons	Zhang et al., 2015
SAG, Purmorphamine	Sonic hedgehog (Shh) activator	Functional neurons	Zhang et al., 2015

Cheng et al. (2015), Zhang et al. (2015) found that ATN transdifferentiation strategies are mediated by epigenetic glial genes silencing and transcriptional activation of neural TFs such as NeuroD1 and NeuroG2. ISX9 (an inducer of neurogenesis) and CHIR99021 were found to mediate the NeuroD1 and NeuroG2 transcriptional activation during neuronal induction based on this finding; specifically, they are thought to be required to activate neuron-specific genes (Li et al., 2015). In contrast, the BET bromodomain inhibitor I-BET151 is a small core molecule required to inhibit endogenous fibroblast fate-determining procedures, possibly by inhibiting BRD4, a member of the BET family (Wu et al., 2015). Furthermore, adding I-BET151 increases the efficiency of neuronal transdifferentiation, indicating that the chemical effectively disrupts the core transcriptional network of fibroblasts (Li et al., 2015). Therefore, we suspected that I-BET151 exerts a similar mechanism to inhibit astrocyte-specific genes. Finally, Y-27632 is a small molecule known as a Rho-associated kinase (ROCK) inhibitor that helps maintain neuronal survival and improves the efficiency of direct chemical reprogramming (Xi et al., 2013; Babos and Ichida, 2015; Zhang et al., 2015).

In summary, using small molecules is a promising approach for facilitating the induction of cellular transdifferentiation into neurons. It is important to fully understand the molecular mechanisms regulated by each molecule and modulate the strength of signaling pathways to generate more precise and effective transdifferentiation strategies.

## 5. Transdifferentiation of neuronal subtypes

The nervous system has a remarkable diversity and heterogeneity of neuronal subtypes. Subtype dysfunction is a key factor in symptoms associated with neurological disorders (Lawler et al., 2020). Induction of target neuronal subtypes more conducive to ameliorating neural disease may be the driving force behind the development of future transdifferentiation strategies. Thus, we summarized the conditions and factors that may be required to induce different neuronal subtypes. First, the main drivers [such as miR-124, miR-9/9*, and PTBP1 (Zhou et al., 2020)] and reinforcing factors [e.g., Nurr1 (Mattugini et al., 2019), Myt1l, and the anti-apoptotic gene Bcl-2/xl (Gascón et al., 2016)] that induce cellular transdifferentiation are not sufficient to specify specific neuronal subtypes. However, they can induce cells with pan-neuronal properties without subtype specification (Cates et al., 2021). Second, the participation of subtype-specifying factors is required to complete the induction of neurons with accurate subtype characteristics (Victor et al., 2014); specifically, they need to be allowed to enter their targets in the transdifferentiation process to function as a specified neuronal subtype because subtype-specifying factors usually have no pioneering activity (Cates et al., 2021). For example, co-expression of the pioneer factor Ascl1 with Brn2 and Myt1l is sufficient to induce dopaminergic neurons *in vitro* and *in vivo*, showing that Brn2 and Myt1l may be specific factors for dopaminergic neuronal subtypes (Pfisterer et al., 2011; Torper et al., 2013; Liu et al., 2015). Brn2, Dlx1/2, Lmx1a, Isl1, and Lhx3 (Hester et al., 2011; Arenas et al., 2015; Pla et al., 2018; Wu et al., 2020), all of which possess neural subtype specification capabilities (Figure 2). In summary, the association between subtypes and SCI can be highlighted in two key points. Firstly, regulating the ratio of glutamatergic and GABAergic neurons in the injured environment is essential. Secondly, there is a need to replenish injured or lost motoneurons in the spinal cord. This study has identified specific factors crucial in specifying distinct neuronal subtypes, particularly through transdifferentiation strategies.

### 5.1. Glutaminergic neurons and GABAergic neurons

Chen et al. (2018) identified spinal cord inhibitory intermediate neurons as barriers that limit the integration of descending inputs into relay circuits after injury. The neurotransmitter phenotypes of specific excitatory intermediate neurons (including glutamatergic neurons) form the basis for motor function recovery after severe SCI (Bertels et al., 2022). Hence, the recovery of spinal cord function may be associated with a decrease in inhibitory GABAergic intermediate neurons and an increase in excitatory glutamatergic neurons (Patel et al., 2021). However, in a motor deficit experiment, the results highlighted the therapeutic potential of spinal GABAergic neurons in SCI (Gong et al., 2021). This finding shows that the level of GABAergic neurons was not as low as possible but achieved a proper balance with excitatory neurons to restore spinal cord function.

The forced expression of different TFs allows for astrocytes’ selective production of glutamatergic or GABAergic neurons *in vivo* (Heinrich et al., 2010). For instance, NeuroG2 has been shown to reprogram non-neuronal cells into glutamatergic neurons in the cortex, and thus NeuroG2 appears to be the main driver (Grande et al., 2013). However, further studies are needed to determine whether NeuroG2 plays a similar role in SCI models. Dlx2 is a TF that plays an important role in the specification and maturation of GABAergic neurons during the development of the central nervous system (Pla et al., 2018). Therefore, in the spinal cord or striatum, an attempt could be made to increase the proportion of GABAergic neurons by combining NeuroD1 with Dlx2, compared with NeuroD1 alone, to produce more glutamatergic neurons. The above data are instructive for matching the damage of different neuronal subtypes with corresponding transdifferentiation protocols.

Although astrocytes can be transdifferentiated into different neuronal subtypes (mainly including glutamatergic and dopaminergic neurons) by a variety of small molecule protocols *in vitro* experiments (Gao et al., 2017; Rivetti di Val Cervo et al., 2017), only a few studies have observed transdifferentiation of astrocytes *in vivo*. Specifically, a small-molecule cocktail (FICBY) induces ATN transformation *in vivo*. Upon analysis, a high proportion of ciNs with glutamatergic neuronal characteristics (72.8%) was observed, which occurred mainly in the cortex (Ma et al., 2021). Interestingly, astrocytes from the cortex, cerebellum, and spinal cord exhibit different biological heterogeneity, limitations, and susceptibilities to neuronal transdifferentiation (Hu et al., 2019). This suggests that different combinations of small molecules affect subtypes and that different regional environments may also have a guiding role in the subtype of ciN. To date, most studies have shown that glutamatergic neurons are the main neural subtype induced by most small molecule protocols (Zhang et al., 2015; Yin et al., 2019). However, there is no evidence for the induction of glutamatergic neurons in SCI, highlighting the need to further search for specific neuronal subtypes in the spinal cord.

### 5.2. Spinal motor neurons

Replenishing injured or lost spinal cord motor neurons is an effective strategy for promoting neural reconstruction and may contribute to improving injured spinal cord function (Bazarek et al., 2022). The forced expression of seven TFs (Ascl1, Brn2, Myt1l, Lhx3, Hb9, Isl1, and NeuroG2) transdifferentiates mouse fibroblasts into induced motor neurons (iMNs). The resulting iMNs exhibit electrophysiological activity, synaptic function, *in vivo* implantation capacity, and sensitivity to disease stimuli (Son et al., 2011). Another study found that three TFs, NeuroG2, Isl1, and Lhx3, effectively programmed spinal cord motor neurons when expressed in differentiated mouse embryonic stem cells (ESCs) (Mazzoni et al., 2013). Therefore, it was hypothesized that Isl1 and Lhx3, present in both studies, might be inducers of spinal cord motor neurogenesis. Isl1 and Lhx3 were later found to induce functional neurons with complex morphologies when co-expressed with miR-9/9*-124 in donor adult fibroblasts. They also specified miR-9/9*-124 to convert neurons into motor neurons by activating a core gene regulatory network (Abernathy et al., 2017). Therefore, this confirmed the hypothesis that Isl1 and Lhx3 selectively drive the conversion of cells into iMNs. Importantly, small molecules also play a guiding role in iMNs. For example, a chemical cocktail (Kenpaullone, Forskolin, Y-27632, Purmorphamine, and Retinoic acid) directly reprogrammed spinal cord astrocytes into iMNs in a mouse model of amyotrophic lateral sclerosis; This perfectly demonstrates the *in vivo* transdifferentiation of reactive astrocytes to neurons under pathophysiological states (Zhao et al., 2020). Although there are no reports of successful induction of iMNs in SCI, the above study not only implies the availability of small molecules for the induction of functional motor neurons in the spinal cord but also illustrates the feasibility of transdifferentiation of resident cells in the injury microenvironment.

### 5.3. Functional neuronal subtypes can be derived from human cells

Although many transdifferentiating factors still do not work on human cells, some exciting findings have achieved a leap forward in human cells. Table 3 summarizes the transdifferentiating schemes currently capable of application in human cells. It is easy to see that transdifferentiation of neural subtypes can be achieved in human cells and that these neural subtypes possess some desirable functional properties. However, transdifferentiation experiments in human cells are limited to *in vitro* experiments. Therefore, the following aspects should be further discussed and resolved to recognize the translation of current research results and their real-life applicability to humans. First, small molecules, as relatively efficient and safe means of delivery, need more evidence from *in vivo* experiments to support their expanded applications. Second, genealogical tracing will be necessary to address the skepticism of *in situ* transdifferentiation results *in vivo*. Crucially, the reproducibility of conclusions in the primate or human brain is necessary. Finally, the criteria for determining successful *in situ* transdifferentiation of neurons need to be established as the vital answer to the reliability of transdifferentiation.

**TABLE 3:** Advances and limitations of transdifferentiation in human cells.

Transdifferentiation factors		References	Animal cells		Human cells		Advances and limitations compared to animal cells
			Starter cell	Targeted cells	Starter cell	Targeted cells	
TFs	Ascl1, Brn2, Myt1l, and NeuroD1	Vierbuchen et al., 2010; Pang et al., 2011	Fibroblasts (No NeuroD1 involved)	Glutamatergic neurons and GABAergic neurons	Fibroblasts	Glutamatergic neurons	Higher purity of functional neurons; Requiring longer culture periods to develop synaptic activity.
	Ascl1, Brn2, Myt1l, Lhx3, Hb9, Isl1, Ngn2, and NeuroD1	Son et al., 2011	Fibroblasts (No NeuroD1 involved)	Motor neurons	Fibroblasts	Motor neurons	Lower transdifferentiation efficiency
	Oct4 and Lhx3	Lee et al., 2020	N/D	N/D	Fibroblasts	Motor neurons	Promoting motor function recovery after transplanting in rodent SCI models; Tumorigenicity from undifferentiated cells limits clinical translation.
	NeuroD1	Guo et al., 2014	astrocytes	Glutamatergic neurons	Astrocytes	Glutamatergic neurons	No directed experiments demonstrated that the functional neurons were derived from astrocytes rather than neurons *in situ*.
	ASCL1 and SOX2	Karow et al., 2018	N/D	N/D	Pericytes	Functional neurons	Pericyte heterogeneity limits their ability to transdifferentiate.
Small molecules	Small molecules (LSTTCVDSP)	Zhang et al., 2015	N/D	N/D	Astrocytes	Glutamatergic neurons	Successfully inducing functional neurons; This protocol failed in animal use.
	Small molecules (VCRFBI)	Gao et al., 2017	N/D	N/D	Astrocytes	Glutamatergic neurons	Inducing functional neurons; No evidence for reprogramming resident astrocytes into neurons *in vivo*.
	Small molecules (KFYPR)	Qin et al., 2018	Fibroblasts	Motor neurons	Fibroblasts	Motor neurons	Neurons induced by this protocol may not survive and mature in the long-term *in vivo* studies.
	Small molecules (KFYPR)	Zhao et al., 2020	Astrocytes	Motor neuron-like cells	Astrocytes	Human motor neuron-like cells	Inducing motor neurons with low survival and possible toxicity *in vivo*.
	Small molecules (CLRDPAI and FYDPAPP)	Yang et al., 2019	N/D	N/D	Fibroblasts	Glutamatergic neuron-like cells	Reprogramming with a lower efficiency
	Small molecules (DAPT, CHIR99021, SB431542, and LDN193189)	Yin et al., 2019	Astrocytes	Neurons	Astrocytes	Glutamatergic neurons	Higher purity of functional neurons; Inability to maintain a constant concentration of small molecules in the brain.
Combinations	miR-124, miR-9/9*, Isl1 and Lhx3	Abernathy et al., 2017	N/D	N/D	Fibroblasts	Motor neurons	A highly pure population of induced human spinal cord motor neurons; No data demonstrate its function *in vivo*.
	TFs (Ngn2, Sox11, Isl1, Lhx3) and small molecules (kenpaullone, forskolin and dorsomorphin)	Liu et al., 2016	N/D	N/D	Fibroblasts	Motor neurons	Small molecules combined with TFs greatly improves the function of iNs; The function was only evaluated in cellular experiments.
	TF (NeuroD1, Ascl1, Lmx1a); miR218 and small molecules (SB431542, LDN193189)	Rivetti di Val Cervo et al., 2017	Astrocytes (no small molecules involved)	Dopaminergic neurons	Astrocytes	Dopaminergic neurons	Challenges to safety and efficacy; Lacks a system to selectively target human astrocytes *in vivo*.

CLRDPAI (CHIR99021, LDN193189, RG108, Dorsomorphin, P7C3-A20, A83-01, and ISX9); FYDPAPP (Forskolin, Y27632, DAPT, PD0325901, A83-01, Purmorphamine, and P7C3-A20); iNs, induced neurons; KFYPR (Kenpaullone, Forskolin, Y-27632, Purmorphamine, Retinoic acid); LSTTCVDSP (LDN193189, SB431542, TTNPB, Thiazovivin, CHIR99021, VPA, DAPT, SAG, and Purmorphamine); N/D, no data; TFs, Transcription factors; VCRFBI (Valproic acid, Chir99021, Repsox, Forskolin, i-Bet151, and ISX-9).

## 6. The role of transdifferentiation-derived neurons in SCI

**FIGURE 3:**
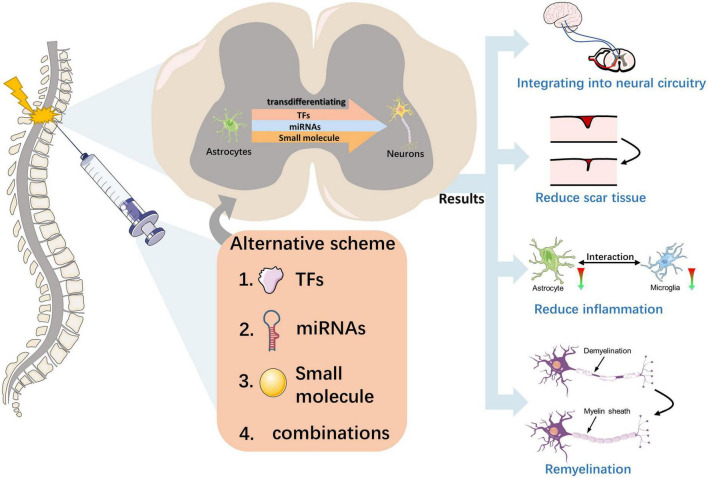
Effect of neuronal transdifferentiation strategies occurring in the injured spinal cord. This figure depicts the transdifferentiation of astrocytes to functional neurons in the injured spinal cord induced by external injection intervention at the macroscopic level with three substances or different combinations of them. The effects of this strategy for the injured spinal cord include the reconstruction of neural circuits, reversal of scar tissue, promotion of myelin regeneration, and alleviation of inflammation, with the ultimate result of promoting the recovery of spinal cord function.

A fundamental question is whether and how these transdifferentiation-derived neurons perform the correct neuronal functions in sensory perception, motor control, and other spinal cord functions (Figure 3). In a study by Zhang et al. (2020), NeuroD1-mediated *in vivo* transformation of ATN reversed glial scars back into nervous tissue in a mouse model of severe puncture injury to the motor cortex. Moreover, ATN transformation rebalanced the neuron-to-glial cell ratio after injury, which is important in restoring normal cortical function. In another study conducted on the mouse spinal cord, the investigation focused on NG2 glial cell-derived neurons that received long-distance projection synapses from the brainstem. This was achieved using a recombinant tracking method based on the rabies virus. The study also examined glial scar formation through GFAP staining, which revealed reduced scar formation (Tai et al., 2021). These findings indicate that circuit reconstruction and scar reduction may contribute to improved functioning after SCI.

Astrocytes and microglia closely interact, and reactive astrocytes can activate microglial activity by secreting pro-inflammatory cytokines (e.g., TGFβ, ATP, and C3) and other factors (Norden et al., 2014; Jo et al., 2017). The pro-inflammatory state induced by M1 microglia and reactive astrocytes often leads to neuroinflammation, which in turn leads to secondary neuronal damage (Hu et al., 2015; Jha et al., 2016). Researchers have investigated the effects of NeuroD1-mediated ATN conversion on microglia and found that decreased reactive astrocytes are accompanied by decreased toxic M1 microglia and neuroinflammation (Zhang et al., 2020). Another study demonstrated that AAV-based NeuroD1 gene therapy could induce cell transformation in brain injury models, leading to decreased astrocytes and potentially alleviating inflammation by reducing the activation of microglia and macrophages (Chen et al., 2020). Even in the SCI model, NeuroD1-mediated gene therapy can induce astrocytes to transform into spinal cord neurons and reduce reactive glial cells, which regulate microglia (Puls et al., 2020). Thus, transdifferentiation strategies can reduce neuroinflammation and improve SCI by reducing the number of toxic astrocytes and M1 microglia.

Overexpression of the TFs OCT4 and KLF4 induces astrocyte reprogramming and inhibits astrocyte migration. Thus, this may be a direct result of reprogramming the resulting NPC to differentiate into specific cell subtypes, likely an oligodendrocyte, thereby promoting myelin regeneration and subsequent recovery of the injured spinal cord (Huang et al., 2020). Another study found that neuregulin-1 is sufficient to transdifferentiate reactive astrocytes into oligodendrocytes and repair SCI through the PI3K-AKT-mTOR signaling pathway (Ding et al., 2021). Therefore, transporting differentiation factors to induce cell transformation into oligodendrocytes to achieve myelin regeneration may be a promising strategy for repairing SCI.

## 7. Conclusion

Some studies have recently shown that many methods, including transdifferentiating factors and small molecules, are powerful tools for manipulating the fate of specific cells. These methods advance neuronal regenerative medicine and help us further explore cell transdifferentiation principles and molecular mechanisms. Simultaneously, small molecules and AAV delivery systems can be delivered safely and *in vivo*, providing a new potential method for clinical application. In addition, the comprehensive protocol of the transdifferentiation strategy combined with rehabilitation has achieved higher effectiveness and timeliness in treating SCI and is expected to achieve higher clinical feasibility in follow-up treatment (Yang et al., 2020).

However, *in vivo* neuronal transdifferentiation of cells (mainly endogenous astrocytes) is still in the early stages of development and has some limitations. For example, neuronal transdifferentiation is relatively inefficient in human cells compared to mouse cells, and *in vivo* applications of transdifferentiation remain limited to animal experiments (Xue et al., 2016). In addition, clinical translation of transdifferentiation protocols is limited due to challenges, including efficiency, scalability, purity, and tumorigenicity from genetic transdifferentiation. As a result, more data are needed to support the clinical translation of this strategy along the path of transdifferentiating regenerative medicine. For instance, more refined behavioral tests could be used so that readers can more intuitively judge the latest progress of transdifferentiation protocols in achieving functional recovery. Despite these challenges, we are confident that in the future, neuronal transdifferentiation will become an effective treatment for SCI.

## Author contributions

Y-MF, W-CC, and W-JZ prepared the manuscript and contributed to writing the original—draft and visualization. Y-SY, YZ, X-LC, and M-QP drew the figures and tables and contributed to the writing—review and editing. SL and H-FH reviewed and finalized the manuscript and contributed to funding acquisition, validation, and resources. All authors had full access to every data in this review and took responsibility for the data’s integrity and the data analysis’s accuracy.
